# Sex-Specific Effects of Buprenorphine on Endoplasmic Reticulum Stress, Abnormal Protein Accumulation, and Cell Loss After Pediatric Mild Traumatic Brain Injury in Mice

**DOI:** 10.1089/neur.2023.0051

**Published:** 2023-08-30

**Authors:** Megan B. Faulkner, Mariam Rizk, Zahraa Bazzi, Robert C. Dysko, Zhi Zhang

**Affiliations:** ^1^ Department of Natural Sciences, University of Michigan–Dearborn, Dearborn, Michigan, USA.; ^2^ Unit for Laboratory Animal Medicine, University of Michigan–Ann Arbor, Ann Arbor, Michigan, USA.

**Keywords:** buprenorphine, ER stress, neuroinflammation, opioid receptor, pediatric, traumatic brain injury

## Abstract

Traumatic brain injury (TBI) in children often leads to poor developmental outcomes attributable to progressive cell loss caused by secondary injuries, including endoplasmic reticulum (ER) stress. Buprenorphine (BPN) is commonly used in children for pain management; however, the effects of BPN on ER stress in the pediatric population are still inconclusive. This study investigated the sex-specific effects of BPN on ER stress, abnormal protein accumulation, and cell loss in a mouse impact acceleration model of pediatric TBI. On post-natal day 20–21 (P20–21), male and female littermates were randomized into sham, TBI + saline and TBI + BPN groups. BPN (0.075 mg/kg) was administered to TBI + BPN mice at 30 min after injury and then every 6–12 h for 2 days. The impact of BPN was evaluated at 1, 3, and 7 days post-injury. We found that TBI induced more prominent ER stress pathway activation at 1 and 3 days post-injury in males, compared to females, whereas abnormal protein accumulation and cell loss were more severe in females at 7 days post-injury, compared with males. Although BPN partially ameliorated abnormal protein accumulation and cell loss in both males and females, BPN only decreased ER stress pathway activation in males, not in females. In conclusion, BPN exhibits sex-specific effects on ER stress, abnormal protein accumulation, and cell loss in a time-dependent manner at the acute phase after pediatric TBI, which provides the rationale to assess the potential effects of BPN on long-term outcomes after pediatric TBI in both males and females.

## Introduction

Traumatic brain injury (TBI) remains a leading cause of morbidity and mortality in children and poses major concerns for public health.^1,2^ Infants and toddlers (0–4 years of age) are at high risk of poor developmental outcomes, such as impaired communication and problem solving,^3^ even after mild TBI.^4^ TBI induces endoplasmic reticulum (ER) stress,^5–12^ which contributes to neuronal loss,^13^ abnormal protein accumulation,^6,14^ tauopathy, and cognitive deficits.^15^ Upon ER stress, unfolded protein response (UPR) is activated to restore ER homeostasis.^16,17^ UPR consists of three major signaling cascades: inositol requiring enzyme 1 (IRE1), activating transcription factor 6 (ATF6), and double-stranded RNA-activated protein kinase-like endoplasmic reticulum kinase (PERK).^18,19^ IRE1 activation induces the unconventional splicing of transcription factor Xbox binding protein 1 (XBP1) to XBP1s (spliced X-box binding protein 1), which increases protein folding and ER biogenesis to restore ER homeostasis.^16,17,20–22^ PERK activation leads to phosphorylation of eukaryotic translation initiation factor-2α (eIF2α) and upregulation of activation transcription factor 4 (ATF4), which lead to greater ER protein folding capacity.^23,24^ ATF6 promotes protein folding and ER-associated degradation.^20^

Although the primary function of UPR is to restore ER homeostasis, unmitigated persistent UPR activation leads to abnormal protein accumulation (e.g. beta-amyloid [Aβ] and phospho-Tau [p-tau])^25–28^ and cell death by the IRE1/TRAF2 (activation stimulates tumor necrosis factor receptor-associated factor 2)/ASK1 (apoptosis signal-regulating kinase)^29^ and PERK/CHOP (C/EBP-homologous protein) pathways.^30^

Buprenorphine (BPN), a synthetic opioid, has been used for pain management among children and adults with TBI^31^ and in pre-clinical animal TBI models.^32^ BPN is a partial agonist of μ-opioid receptors (MORs), and an antagonist of δ-opioid receptors and κ-opioid receptors (KORs).^31^ Studies have also shown that opioids can inhibit the innate and adaptive immune systems^33^ and exert protective effects in response to cytotoxic insults by reducing calcium overload and suppressing ER stress sensors.^34,35^ BPN treatments can limit chemokine (C-C motif) ligand 2–mediated monocyte transmigration^36^ and alter microglial and astrocytes mediated neuroinflammation after TBI.^37^ Our previous study indicates that BPN normalizes MOR expression in white matter astrocytes, improves sensorimotor function, and decreases oxidative stress after pediatric TBI.^38^ To date, no study has investigated the sex-specific effects of BPN on ER stress, abnormal protein accumulation, and cell death after pediatric TBI. Here, we investigated the sex-specific effects of BPN on ER stress pathways, Aβ and tau accumulation, and cell loss after pediatric TBI in both males and females.

## Methods

### Animals

Male (M) and female (F) C57BL/6 mice (2–3 months of age; The Jackson Laboratory, Bar Harbor, ME) were in-house bred. All pups were delivered naturally and remained with their mother after birth until weaning. All animals (2–5 mice per cage) were housed under standard housing conditions (20°C–22°C, 40–60% relative humidity, and a 12-h light/dark cycle) with free access to food and water. All experimental procedures were approved by the Institutional Animal Care and Use Committee (IACUC) of the University of Michigan.

### Impact acceleration model of traumatic brain injury and buprenorphine administration

On post-natal days 20–21 (P20-21, equivalent to 2–3 years of age in humans),^39^ animals from the same litter were randomized into sham (*n* = 64; 31M/33F), TBI + saline (*n* = 74; 37M/37F), and TBI + BPN (*n* = 73; 34M/39F) groups. Animals in the TBI + saline and TBI + BPN groups underwent TBI as previously described.^38^ BPN was diluted in 0.9% NaCl (sterile) to a concentration of 0.01 mg/mL. The TBI + BPN group received intraperitoneal injections of BPN (0.075 mg/kg) at 30 min after injury and then every 6–12 h for 2 days as previously published.^38^ The TBI + saline group received the same volume of saline at 30 min after surgery and then every 6–12 h for 2 days. The sham group did not receive any intervention. All animals were closely monitored as per IACUC guidelines.

### RNA isolation and quantitative real-time polymerase chain reaction

mRNA expression of IRE-1α, XPB1s, PERK, ATF6, eIF2α, ATF4, TRAF2, ASK1, and CHOP were measured at the site of injury at 1 day (sham: *n* = 13, 6M/7F; TBI + saline: *n* = 12, 6M/6F; TBI + BPN: *n* = 13, 6M/7F), 3 days (sham: *n* = 10, 5M/5F; TBI + saline: *n* = 18, 8M/10F; TBI + BPN: *n* = 16, 8M/8F), and 7 days (sham: *n* = 11, 5M/6F; TBI + saline: *n* = 14, 8M/6F; TBI + BPN: *n* = 14, 5M/9F) post-injury. Brain tissues (approximately between bregma +2 mm and bregma −1 mm) were microdissected for RNA isolation as previously described.^38^ Total RNA was extracted using TRIzol (Sigma-Aldrich, St. Louis, MO) and were quantified using the Nanodrop ND-2000 Spectrophotometer (ThermoFisherScientific, Waltham, MA). Single-stranded complementary DNA (cDNA) was reverse transcribed from RNA using the High-Capacity cDNA Reverse Transcription Kit with RNase inhibitor (ThermoFisherScientific). Primers were custom designed (Table 1) and ordered from Integrated DNA Technology (Coralville, IA). Quantitative real-time polymerase chain reaction (qPCR) was performed with iTaq(tm) Universal SYBR(R) Green Supermix (Bio-Rad Laboratories, Hercules, CA), and the comparative threshold cycle method was used to assess differential gene expressions.^38^

**Table 1.** Primers for qPCR

Gene	Forward primer	Reverse primer
*Ire1a*	CTGTGGTCAAGATGGACTGG	GAAGCGGGAAGTGAAGTAGC
*Xbp1s*	GAGTCCGCAGCAGGTG	GTGTCAGAGTCCATG GGA
*Perk*	TCAAGTTTCCTCTACTGTTCACTCA	CGGGAAAC TCCAAGTTCTCA
*Atf6*	CTGGGCTCGGTAGTTTGTATC	AGACCTGAATGGCTGCTTAC
*eIF2α*	ATGCAGAAGTGGATGGAGATG	GGCTGCTTCGTACTCCTTAAA
*Atf4*	TATGACCCACCTGGAGTTAGT	CTAGTGGCTGCTGTCTTGTT
*Traf2*	CAGTGAGGAGCTGTCACATTAG	GTGATGGTGATTGGCTCTAGG
*Ask1*	GACAAGAGAGCCTGTGCTAATG	TCCAGTCGAGAGAGCTGAAA
*Chop*	TATCTCATCCCCAGGAAACG	GGG CACTGACCACTCTGTTT
*Gapdh*	AACAGCAACTCCCACTCTTC	CCTGTTGCTGTAGCCGTATT

qPCR, quantitative real-time polymerase chain reaction.

### Immunohistochemistry

Brains were post-fixed in 10% formalin for 48 h and cryoprotected in 30% sucrose (in phosphate-buffered saline [PBS]). Coronal sections (20 μm, 1:6 series) were prepared on a cryostat (Leica Microsystems, Buffalo Grove, IL). For double labeling of CHOP and p-tau, brain sections were incubated overnight at 4°C with mouse anti-CHOP (growth arrest- and DNA damage-inducible gene 153; 1:250, GeneTex, Inc., Irvine, CA) and rabbit anti-p-tau (1:250; ThermoFisherScientific). For double labeling of Aβ and Fluoro-Jade C (FJC),^40,41^ brain sections were washed three times in PBS for 5 min each, incubated in 0.001% FJC for 10 min, washed six times in PBS for 15 min each, and incubated with rabbit anti-Aβ (1:250; ThermoFisherScientific) overnight at 4°C. After wash, brain sections were incubated with fluorescent secondary antibodies (1:250; Life Technologies, Carlsbad, CA) for 2 h at room temperature. Slides were dried and cover-slipped with fluorescent mounting medium with 4′,6-diamidino-2-phenylindole (DAPI; Sigma-Aldrich).

### Histology quantification

Histological quantification was performed at 1 day (sham: *n* = 10, 5M/5F; TBI + saline: *n* = 10, 5M/5F; TBI + BPN: *n* = 10, 5M/5F), 3 days (sham: *n* = 10, 5M/5F; TBI + saline: *n* = 10, 5M/5F; TBI + BPN: *n* = 10, 5M/5F), and 7 days (sham: *n* = 10, 5M/5F; TBI + saline: *n* = 10, 5M/5F; TBI + BPN: *n* = 10, 5M/5F) post-injury. Images were acquired using a Nikon Eclipse TS2R fluorescent microscope (Nikon Inc., Melville, NY) with same camera settings. All slides and images were coded, and the analysis was performed with personnel blinded to the experiments. Images (40 × , five images per animal) were randomly acquired from the injured cortex (or the matching area in the sham). Expression and distribution of CHOP, p-tau, Aβ, and FJC were evaluated as the percentage of area using particle analysis function in Fiji ImageJ software (National Institutes of Health, Bethesda, MD) as previously described.^28,38,42^ Percentages of CHOP^+^p-Tau^+^ and FJC^+^Aβ^+^ were measured using Fiji ImageJ (National Institutes of Health) as previously described.^28,38,42^ Percentages of CHOP^+^p-Tau^+^ and FJ-C^+^Aβ^+^ cells were calculated as follows:

$$\begin{array}{cc} Thepercentage & =thenumberof\left(CHOP^{+}p-Tau^{+}\right)∕\\ & \left(thenumberofCHOP^{+}cells\right)\times 100\%, \end{array}$$

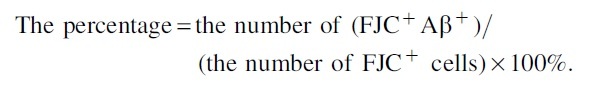


### Statistical analysis

Data were analyzed using GraphPad Prism 6 (Version 6.04; CA, USA). All data are presented as mean ± standard error of the mean. The D'agostino and Pearson omnibus normality test was used for normality measurement. The Student's *t*-test (two-tailed) or Mann-Whitney U test (two-tailed) were used for two-group comparisons. One-way analysis of variance and Bonferroni's *post hoc* tests were used for multiple group comparisons. Statistical significance was set at *p* < 0.05 for all analyses.

## Results

### Sex-specific effects of buprenorphine on endoplasmic reticulum stress pathways

**FIG. 1.**
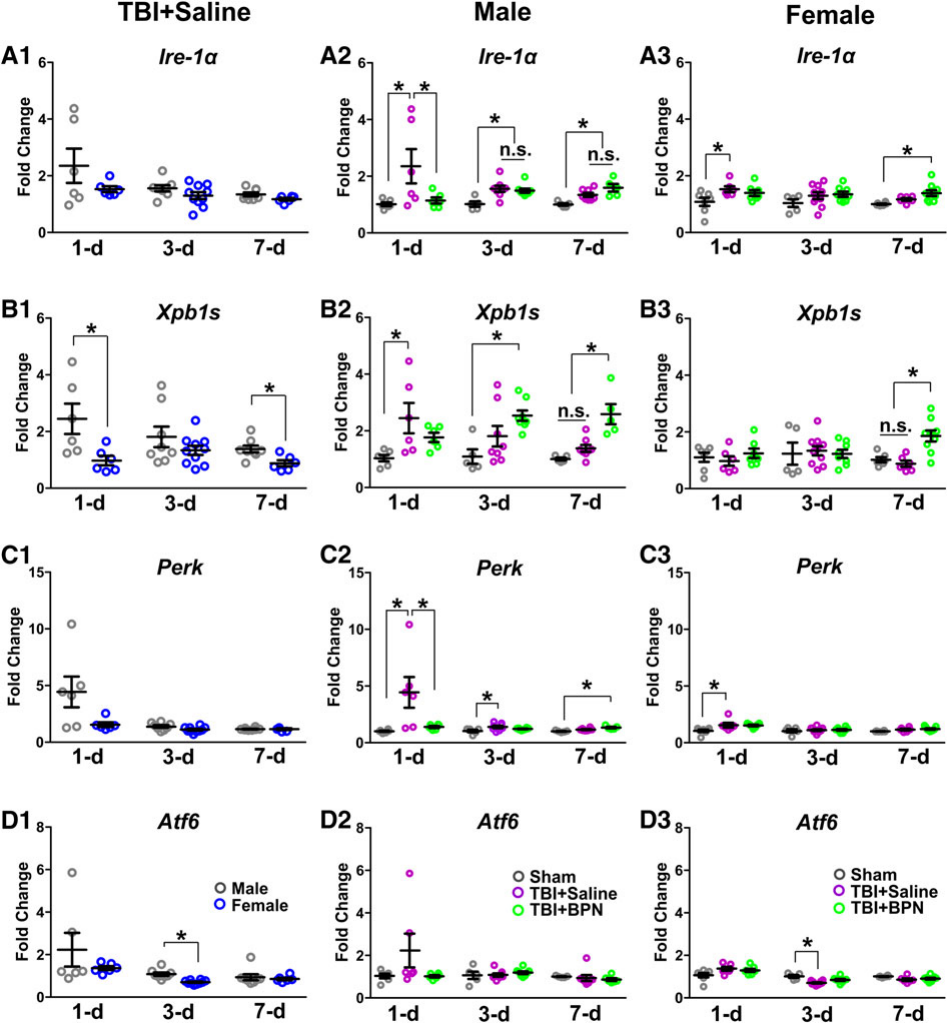
mRNA expression of IRE-1α, XPB1s, PERK, and ATF6 in the injured brain regions at 1, 3, and 7 days post-injury. (**A1–A3**) The mRNA expression of IRE-1α in TBI + saline males versus females (A1), males (A2), and females (A3). (**B1–B3**) The mRNA expression of XPB1s in TBI + saline males versus females (B1), males (B2), and females (B3). (**C1–C3**) The mRNA expression of PERK in TBI + saline males vs. females (C1), males (C2), and females (C3). (**D1–D3**) The mRNA expression of ATF6 in TBI + saline males versus females (D1), males (D2), and females (D3). *p < 0.05. ATF6, activating transcription factor 6; BPN, buprenorphine; IRE-1α, inositol requiring enzyme 1; mRNA, messenger RNA; n.s., no significance; PERK, protein kinase-like endoplasmic reticulum kinase; TBI, traumatic brain injury; XPB1s, spliced X-box binding protein 1.

We first compared the messenger RNA (mRNA) expression of IRE-1α, XPB1s, PERK, and ATF6 between male and female TBI + saline mice. We found that XBP1s significantly decreased at 1 day (*p* = 0.03) and 7 days (*p* = 0.01; Fig. 1B1) and ATF6 significantly decreased at 3 days post-injury (*p* = 0.0001) in female TBI + saline mice (Fig. 1D1). There was no difference in IRE-1α and PERK expressions (Fig. 1A1,C1). In males, IRE-1α significantly increased in TBI + saline at 1 day post-injury (*F* = 4.3, *p* = 0.03; vs. sham and TBI + BPN) and significantly increased in both the TBI + saline and TBI + BPN groups at 3 days (*F* = 7.7, *p* = 0.004; vs. sham) and 7 days (*F* = 9.9, *p* = 0.002; vs. sham) post-injury (Fig. 1A2). XPB1s significantly increased in TBI + saline at 1 day post-injury (*F* = 4.6, *p* = 0.03; vs. sham) and significantly increased in TBI + BPN at 3 days (*F* = 5.5, *p* = 0.01; vs. sham) and 7 days post-injury (*F* = 15.5, *p* = 0.0002; vs. sham and TBI + saline; Fig. 1B2). PERK significantly increased in TBI + saline at 1 day (*F* = 5.7, *p* = 0.01; vs. sham and TBI + BPN) and 3 days post-injury (*F* = 3.7, *p* = 0.04; vs. sham) and significantly increased in TBI + BPN at 7 days post-injury (*F* = 10.6, *p* = 0.001; vs. sham; Fig. 1C2). There was no change in ATF6 expression (Fig. 1D2).

In females, IRE-1α significantly increased in TBI + saline at 1 day post-injury (*F* = 6.3, *p* = 0.04; vs. sham) and significantly increased in the TBI + BPN at 7 days post-injury (*F* = 5.9, *p* = 0.01; vs. sham; Fig. 1A3). XPB1s significantly increased in TBI + BPN at 7 days post-injury (*F* = 11.5, *p* = 0.0006; vs. sham and TBI + saline; Fig. 1B3). PERK significantly increased in TBI + saline at 1 day post-injury (*F* = 4.7, *p* = 0.02; vs. sham; Fig. 1C3). ATF6 significantly decreased in TBI + saline at 3 days post-injury (*F* = 11.0, *p* = 0.0006; vs. sham; Fig. 1D3).

**FIG. 2.**
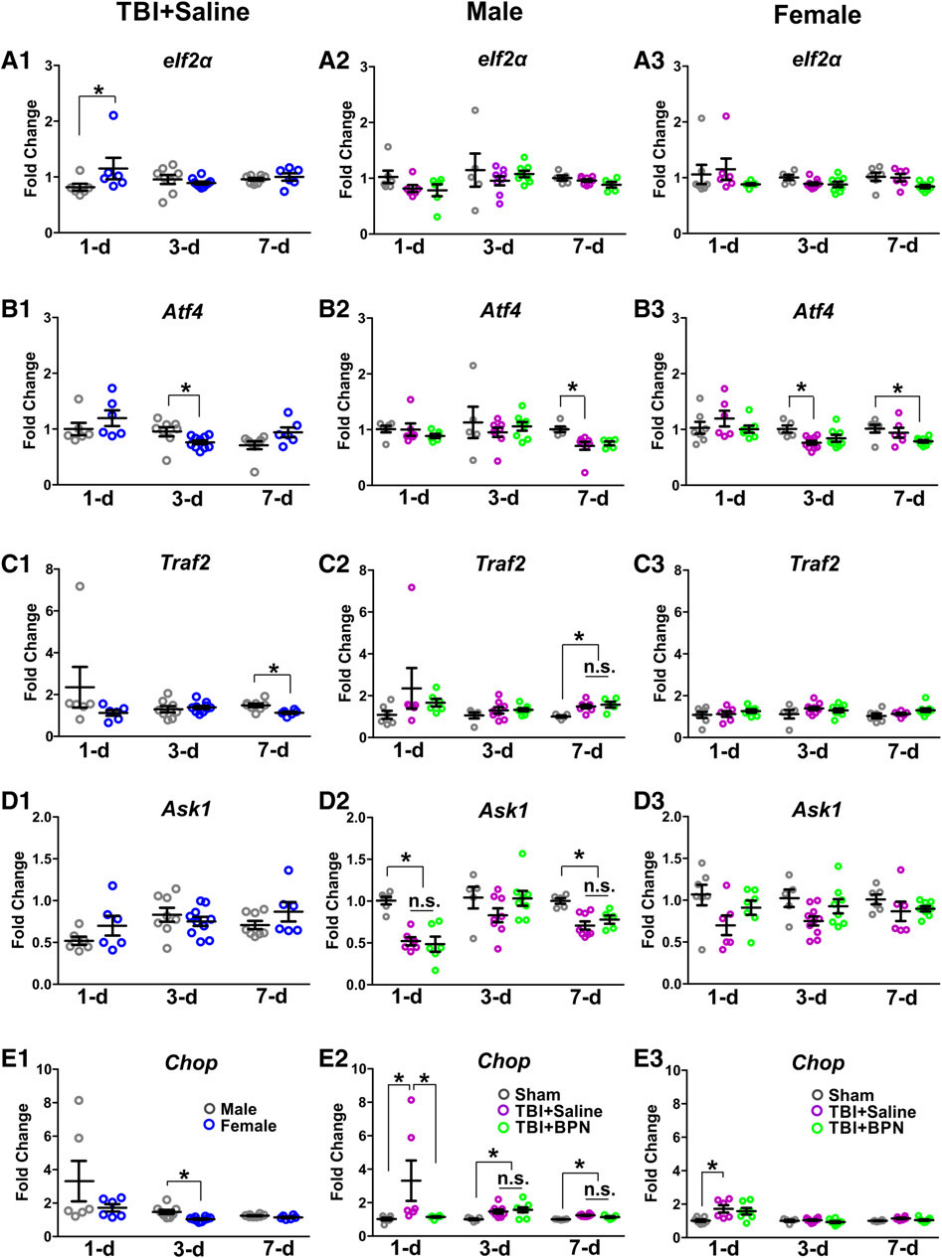
mRNA expression of eIF2α, ATF4, TRAF2, ASK1, and CHOP in the injured brain regions at 1, 3, and 7 days post-injury. (**A1–A3**) mRNA expression of eIF2α in TBI + saline males versus females (A1), males (A2), and females (A3). (**B1–B3**) mRNA expression of ATF4 in TBI + saline males versus females (B1), males (B2), and females (B3). **(C1–C3**) mRNA expression of TRAF2 in TBI + saline males versus females (C1), males (C2), and females (C3). (**D1–D3**) mRNA expression of ASK1 in TBI + saline males versus females (D1), males (D2), and females (D3). (**E1–E3**) mRNA expression of CHOP in TBI + saline males versus females (E1), males (E2), and females (E3). *p < 0.05. ASK1, apoptosis signal-regulating kinase 1; ATF4, activation transcription factor 4; BPN, buprenorphine; CHOP, C/EBP-homologous protein; eIF2α, eukaryotic translation initiation factor-2α; mRNA, messenger RNA; n.s., no significance; TBI, traumatic brain injury; TRAF2, activation stimulates tumor necrosis factor receptor-associated factor 2.

We next investigated the mRNA expression of eIF2α, AFT4, TRAF2, ASK1, and CHOP between male and female TBI + saline mice. We found that eIF2α significantly increased in female TBI + saline mice at 1 day post-injury (*p* = 0.04; Fig. 2A1). ATF4 significantly decreased in female TBI + saline mice at 3 days post-injury (*p* = 0.04; Fig. 2B1). TRAF2 significantly decreased in female TBI + saline mice at 7 days post-injury (*p* = 0.007; Fig. 2C1). There was no significant difference in ASK1 (Fig. 2D1). CHOP significantly decreased in female TBI + saline mice at 3 days post-injury (*p* = 0.002; Fig. 2E1). In males, there was no significant difference in eIF2α expression (Fig. 2A2). ATF4 significantly decreased in TBI + saline at 7 days post-injury (*F* = 5.9, *p* = 0.01; vs. sham; Fig. 2B2). TRAF2 significantly increased in both TBI + saline and TBI + BPN at 7 days post-injury (*F* = 8.6, *p* = 0.003; vs. sham; Fig. 2C2). ASK1 significantly decreased in TBI + saline and TBI + BPN at 1 day (*F* = 19.9, *p* < 0.0001; vs. sham) and 7 days post-injury (*F* = 9.8, *p* = 0.002; vs. sham; Fig. 2D2). CHOP significantly increased in TBI + saline at 1 day post-injury (*F* = 11.1, *p* = 0.004; vs. sham and TBI + BPN) and significantly increased in both the TBI + saline and TBI + BPN groups at 3 days (*F* = 4.2, *p* = 0.03; vs. sham) and 7 days (*F* = 15.5, *p* = 0.0002; vs. sham) post-injury (Fig. 2E2).

In females, ATF4 significantly decreased in TBI + saline at 3 days post-injury (*F* = 5.1, *p* = 0.02; vs. sham) and in TBI + BPN at 7 days post-injury (*F* = 3.9, *p* = 0.04; vs. sham; Fig. 2B3). CHOP significantly increased in TBI + saline at 1 day post-injury (*F* = 4.8, *p* = 0.02; vs. sham; Fig. 2E3). There was no significant difference in eIF2α, TRAF2, and ASK1 expressions (Fig. 2A3,C3,D3).

### Sex-specific effects of buprenorphine on p-tau

**FIG. 3.**
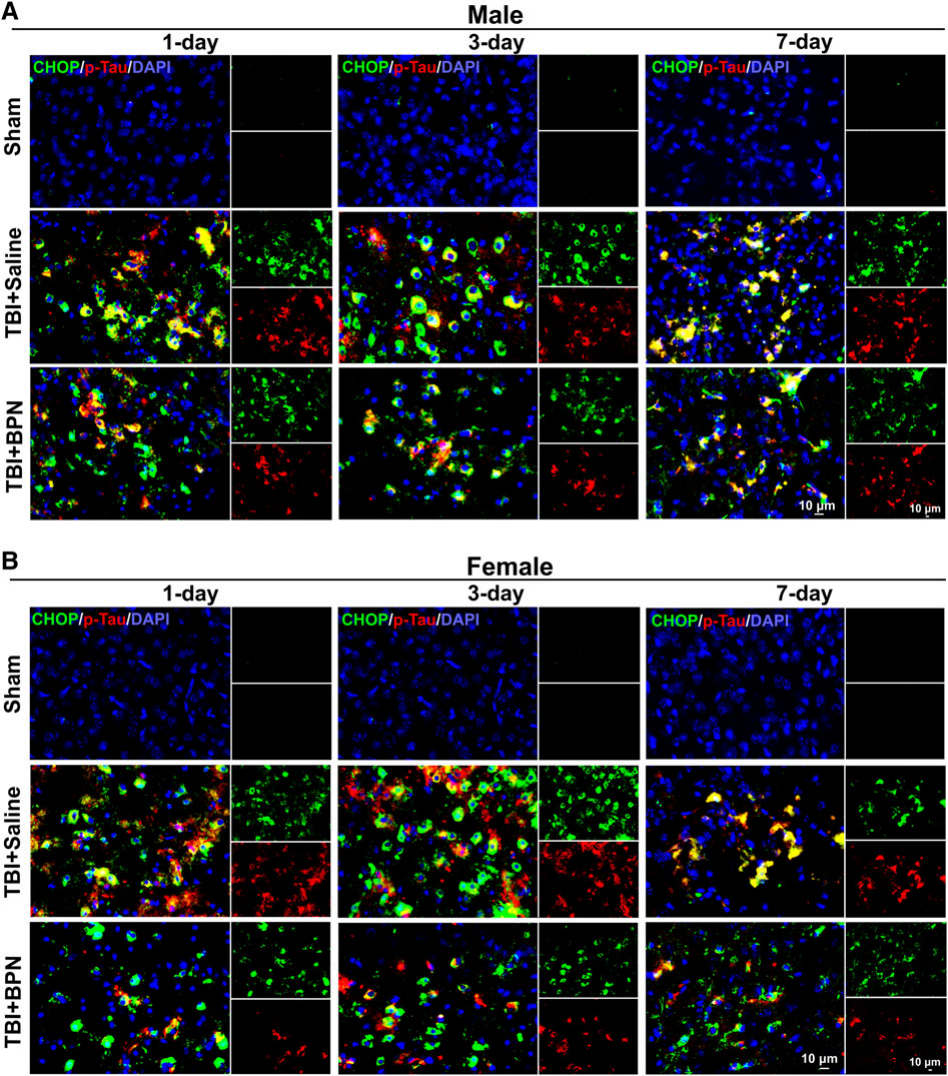
Expression of CHOP and p-tau and the percentage of colocalization of CHOP and p-tau at the cortex of injured brain regions at 1, 3, and 7 days post-injury. (**A**) Representative images of CHOP (green) and p-tau (red) expression at 1, 3, and 7 days post-injury in males. (**B**) Representative images of CHOP (green) and p-tau (red) expression at 1, 3, and 7 days post-injury in females. Nuclei were stained with DAPI (blue). Scale bar, 10 μm. BPN, buprenorphine; CHOP, C/EBP-homologous protein; DAPI, 4′,6-diamidino-2-phenylindole; p-tau, phosphor-tau; TBI, traumatic brain injury.

We first compared the expression of CHOP and p-tau, and the percentage of CHOP^+^p-Tau^+^ cells between male and female TBI + saline mice. There was no significant difference in CHOP and p-tau expressions (Fig. 3 and Fig. 5A1, B1). Percentage of CHOP^+^p-Tau^+^ significantly decreased at 1 day post-injury (*p* = 0.04) and significantly increased at 3 days post-injury (*p* = 0.02) in female TBI + saline mice (Fig. 3 and Fig. 5C1). In males, CHOP significantly increased in both TBI + saline and TBI + BPN at 1 day (*F* = 138.0, *p* < 0.0001), 3 days (*F* = 150.0, *p* < 0.0001), and 7 days (*F* = 80.9, *p* < 0.0001) post-injury, compared with sham (Fig. 3 and Fig. 5A2). p-tau significantly increased in both TBI + saline and TBI + BPN at 1 day (*F* = 61.3, *p* < 0.0001), 3 days (*F* = 58.4, *p* < 0.0001), and 7 days (*F* = 82.0, *p* < 0.0001) post-injury, compared with sham. Moreover, p-tau significantly increased in TBI + saline at 1 and 3 days post-injury, compared with TBI + BPN (*p* < 0.05; Fig. 3 and Fig. 5B2).

The percentage of CHOP^+^p-Tau^+^ significantly increased in both TBI + saline and TBI + BPN at 1 day (*F* = 38.3, *p* < 0.0001), 3 days (*F* = 46.1, *p* < 0.0001), and 7 days (*F* = 178.5, *p* < 0.0001) post-injury, compared with sham. Moreover, the percentage of CHOP^+^p-Tau^+^ significantly increased in TBI + saline at 1 day (*p* < 0.05) post-injury, compared with TBI + BPN (Fig. 3 and Fig. 5C2). In females, CHOP significantly increased in both TBI + saline and TBI + BPN at 1 day (*F* = 124.2, *p* < 0.0001), 3 days (*F* = 75.5, *p* < 0.0001), and 7 days (*F* = 43.1, *p* < 0.0001) post-injury, compared with sham (Fig. 3 and Fig. 5A3). p-tau significantly increased in both TBI + saline and TBI + BPN at 1 day (*F* = 31.1, *p* < 0.0001), 3 days (*F* = 99.5, *p* < 0.0001), and 7 days (*F* = 73.2, *p* < 0.0001) post-injury, compared with sham. Moreover, p-tau significantly increased in TBI + saline at 1, 3, and 7 days post-injury, compared with TBI + BPN (p < 0.05; Fig. 3 and Fig. 5B3). The percentage of CHOP^+^p-Tau^+^ significantly increased in both TBI + saline and TBI + BPN at 1 day (*F* = 39.0, *p* < 0.0001), 3 days (*F* = 57.7, *p* < 0.0001), and 7 days (*F* = 101.7, *p* < 0.0001) post-injury, compared with sham. Moreover, the percentage of CHOP^+^p-Tau^+^ significantly increased in TBI + saline at 1, 3, and 7 days post-injury, compared with TBI + BPN (*p* < 0.05; Fig. 3 and Fig. 5C3).

### Sex-specific effects of buprenorphine on amyloid-beta accumulation and cell loss

**FIG. 4.**
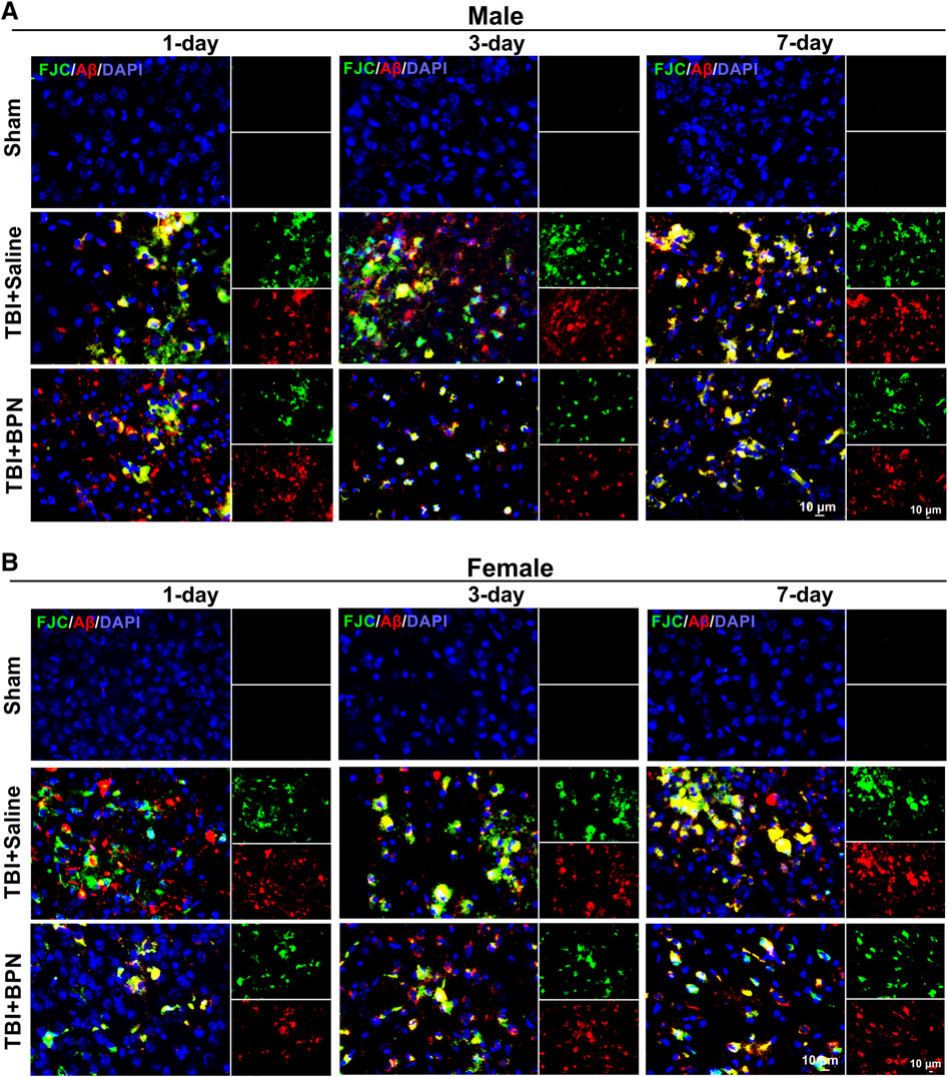
Expression of FJC and Aβ and the percentage of colocalization of FJC and Aβ at the cortex of injured brain regions at 1, 3, and 7 days post-injury. (**A**) Representative images of FJC (green) and Aβ (red) expression at 1, 3, and 7 days post-injury in males. (**B**) Representative images of FJC (green) and Aβ (red) expression at 1, 3, and 7 days post-injury in females. Nuclei were stained with DAPI (blue). Scale bar, 10 μm. Aβ, beta-amyloid; BPN, buprenorphine; DAPI, 4′,6-diamidino-2-phenylindole; FJC, Fluoro-Jade C; TBI, traumatic brain injury.

**FIG. 5.**
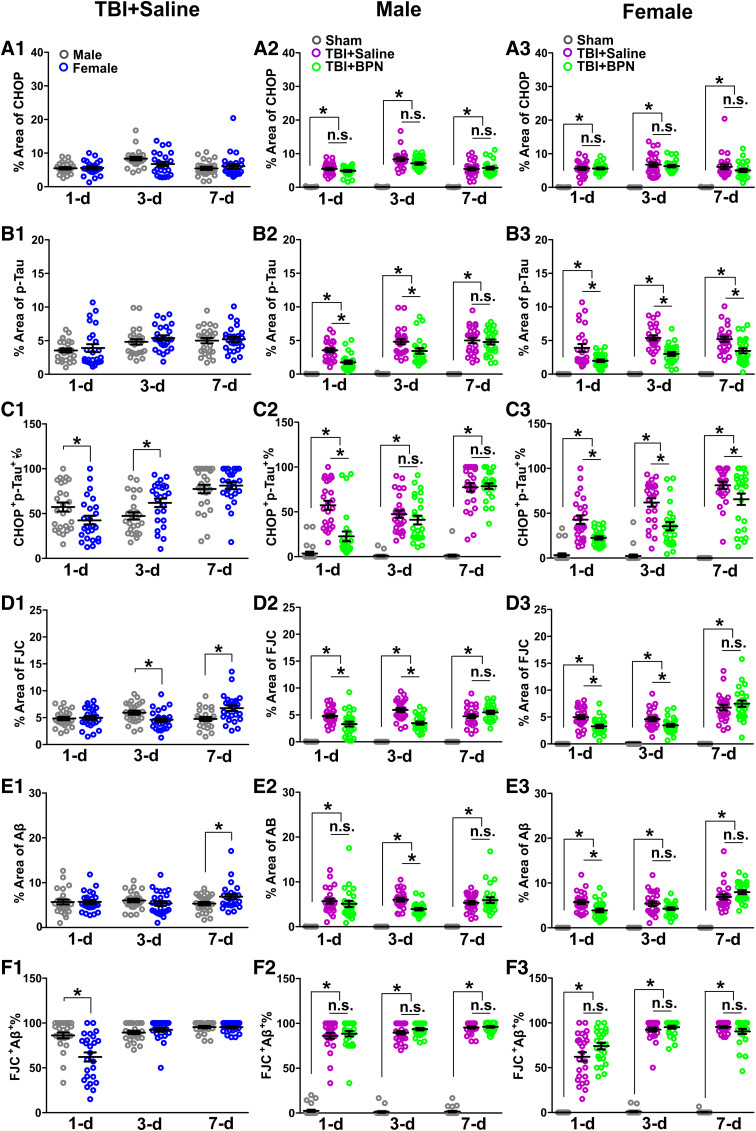
Expression of CHOP and p-tau and the percentage of CHOP^+^p-Tau^+^ cells (**A–C**) and the expression of FJC and Aβ and the percentage of FJC^+^Aβ^+^ cells (**D–F**) at the cortex of injured brain regions at 1, 3, and 7 days post-injury. Protein expressions were measured semi quantitatively using immunohistochemistry antibody staining. (A1–A3) Percentage area of CHOP expression in TBI + saline males versus females (A1), males (A2), and females (A3). (B1–B3) Percentage area of p-tau expression in TBI + saline males versus females (B1), males (B2), and females (B3). (C1–C3) Percentage of CHOP^+^p-Tau ^+^ cells in TBI + saline males versus females (C1), males (C2), and females (C3). (D1–D3) Percentage area of FJC expression in TBI + saline males versus females (D1), males (D2), and females (D3). (E1–E3) Percentage area of Aβ expression in TBI + saline males versus females (E1), males (E2), and females (E3). (F1–F3) Percentage of FJC^+^Aβ ^+^ cells in TBI + saline males versus females (F1), males (F2), and females (F3). *p < 0.05. Aβ, beta-amyloid; BPN, buprenorphine; CHOP, C/EBP-homologous protein; FJC, Fluoro-Jade C; n.s., no significance; p-tau, phosphor-tau; TBI, traumatic brain injury.

We first compared the expression of FJC and Aβ and the percentage of FJC^+^Aβ^+^ cells between male and female TBI + saline mice. FJC significantly decreased at 3 days post injury (*p* = 0.01), but significantly increased at 7 days post-injury (*p* = 0.003) in female TBI + saline mice (Fig. 4 and Fig. 5D1). Aβ significantly increased at 7 days post-injury (*p* = 0.03) in female TBI + saline mice (Fig. 4 and Fig. 5E1). The percentage of FJC^+^Aβ^+^ significantly decreased at 1 day post-injury (*p* = 0.0002) in female TBI + saline mice (Fig. 4 and Fig. 5F1). In males, FJC significantly increased in both TBI + saline and TBI + BPN at 1 day (*F* = 62.9, *p* < 0.0001), 3 days (*F* = 122.3.0, *p* < 0.0001), and 7 days (*F* = 121.5, *p* < 0.0001) post-injury, compared with sham. Moreover, FJC significantly increased at 1 and 3 days post-injury in TBI + saline, compared with TBI + BPN (*p* < 0.05; Fig. 4 and Fig. 5D2).

Aβ significantly increased in both TBI + saline and TBI + BPN at 1 day (*F* = 38.0, *p* < 0.0001), 3 days (*F* = 118.9, *p* < 0.0001), and 7 days (*F* = 48.0, *p* < 0.0001) post-injury, compared with sham. Moreover, Aβ significantly increased in TBI + saline at 3 days post-injury (*p* < 0.05), compared with TBI + BPN (Fig. 4 and Fig. 5E2). The percentage of FJC^+^Aβ^+^ significantly increased in both TBI + saline and TBI + BPN at 1 day (*F* = 343.3, *p* < 0.0001), 3 days (*F* = 1303.0, *p* < 0.0001), and 7 days (*F* = 2868.0, *p* < 0.0001) post-injury, compared with sham (Fig. 4 and Fig. 5F2).

In females, FJC significantly increased in both TBI + saline and TBI + BPN at 1 day (*F* = 84.1, *p* < 0.0001), 3 days (*F* = 82.7.0, *p* < 0.0001), and 7 days (*F* = 83.6, *p* < 0.0001) post-injury, compared with sham. Moreover, FJC significantly increased in TBI + saline at 1 and 3 days post-injury, compared with TBI + BPN (*p* < 0.05; Fig. 4 and Fig. 5D3). Aβ significantly increased in both TBI + saline and TBI + BPN at 1 day (*F* = 74.1, *p* < 0.0001), 3 days (*F* = 71.1, *p* < 0.0001), and 7 days (*F* = 106.4, *p* < 0.0001) post-injury, compared with sham. Moreover, Aβ significantly increased in TBI + saline at 1 day post-injury (*p* < 0.05), compared with TBI + BPN (Fig. 4 and Fig. 5E3). The percentage of FJC^+^Aβ^+^ significantly increased in both TBI + saline and TBI + BPN at 1 day (*F* = 126.4, *p* < 0.0001), 3 days (*F* = 1085, *p* < 0.0001), and 7 days (*F* = 937.9, *p* < 0.0001) post-injury, compared with sham (Fig.4 and Fig. 5F3).

## Discussion

Evidence has shown that ER stress responses are different between male and female.^43,44^ In the present study, we demonstrated that both sexes showed a significant upregulation of ER stress genes; however, the magnitude of gene upregulation was different between males and females in a time-dependent manner. For example, CHOP significantly decreased at 3 days and eIF2α significantly increased at 1 day post-injury in female TBI + saline mice, compared with males. CHOP expression increases after TBI and is a representative feature of ER stress-induced cell death,^45,46^ whereas eIF2α promotes cell survival in response to oxidative stress.^47^ Therefore, the decreased CHOP and increased eIF2α may be partially responsible for the decreased cell loss at 3 days post-injury in the females.

Interestingly, BPN significantly decreased CHOP at 1 day post-injury in male mice, but not in females. These sex differences in BPN might be partially attributable to the differences in opioid receptor expression and distribution.^48^ For example, we have previously demonstrated that MOR expression significantly increased at 1 and 7 days post-injury in the male TBI + saline mice after pediatric TBI, but not in females.^38^ Evidence indicates that MOR activation can decrease ER stress and protect cells from excitotoxicity-induced apoptosis,^35^ whereas KOR activation induces apoptosis through the enhanced ER stress pathway.^49^ BPN can exert its effects through activation of MORs and inhibition of KORs, leading to decreased cell death. In addition, BPN significantly increased XPB1s at 7 days post-injury in both male and female TBI + BPN mice, compared to sham and TBI + saline mice. Evidence indicates that overexpression of XBP1s rescues Aβ neurotoxicity^50^ and ameliorates tauopathy.^51^ Therefore, increased XPB1s can be beneficial for reducing abnormal protein accumulation.

Interestingly, there is a discrepancy between mRNA and protein expression of CHOP. For example, in females, mRNA expression of CHOP was similar among groups at 3 and 7 days post-injury; however, protein expression of CHOP significantly increased in both the TBI + saline and TBI + BPN groups. This indicates that CHOP expression may be regulated transcriptionally and/or mRNA stability.^45^

Studies have demonstrated acute Aβ and tau aggregation and cell loss in both TBI patients and animal models of TBI.^52,53^ Here, we demonstrated that TBI caused a persistent increase of CHOP-, p-tau-, Aβ-, and FJC-positive cells at the injured brain regions. Our results are consistent with a previous study, in which there is a positive correlation between CHOP, Aβ, and p-tau.^6^ Interestingly, FJC and Aβ significantly increased in female TBI + saline mice at 7 days post-injury, compared with males. Studies have shown that females exhibit higher Aβ and tau burden than males, which is significantly associated with poor outcomes.^54–58^ We have previously demonstrated that female TBI + saline mice exhibit significantly increased depressive-like behaviors at 7 days post-injury, compared to males.^38^ These behavioral deficits might be related to the increased Aβ burden and cell loss in females. We further demonstrate that BPN significantly decreased p-tau and FJC at 1 and 3 days post-injury, suggesting a neuroprotective effect against Aβ and p-tau toxicity.^50,51^ Together, our study provides a first line of evidence that BPN reduces ER stress, abnormal protein accumulation, and cell loss in a time- and sex-dependent manner after pediatric TBI. However, the underlying mechanisms of BPN actions need further investigation.

## Conclusion

This study demonstrates that BPN treatment attenuated ER stress pathway activation, reduced Aβ and p-tau accumulation, and decreased cell loss in a time- and sex-dependent manner. Additional studies are warranted to study the long-term effects of BPN in both males and females after pediatric TBI.

## Abbreviations Used

Aβ: beta-amyloid
ASK1: apoptosis signal-regulating kinase 1
ATF4: activation transcription factor 4
ATF6: activating transcription factor 6
BP: buprenorphine
cDNA: complementary DNA
CHOP: C/EBP-homologous protein
DAPI: 4′,6-diamidino-2-phenylindole
eIF2α: eukaryotic translation initiation factor-2α
ER: endoplasmic reticulum
FJC: Fluoro-Jade C
IACUC: Institutional Animal Care and Use Committee
IRE1: inositol requiring enzyme 1
KORs: κ-opioid receptors
MORs: μ-opioid receptors
mRNA: messenger RNA
PBS: phosphate-buffered saline
PERK: protein kinase-like endoplasmic reticulum kinase
p-tau: 24hosphor-Tau
qPCR: quantitative real-time polymerase chain reaction
TBI: traumatic brain injury
TRAF2: activation stimulates tumor necrosis factor receptor-associated factor 2
UPR: unfolded protein response
XBP1: Xbox binding protein 1
XPB1s: spliced X-box binding protein 1

## References

[1] Keenan HT, Bratton SL. Epidemiology and outcomes of pediatric traumatic brain injury. Dev Neurosci 2006;28(4–5):256–263; doi: 10.1159/000094152 16943649

[2] Schneier AJ, Shields BJ, Hostetler SG, et al. Incidence of pediatric traumatic brain injury and associated hospital resource utilization in the United States. Pediatrics 2006;118(2):483–492; doi: 10.1542/peds.2005-2588 16882799

[3] Keenan HT, Presson AP, Clark AE, et al. Longitudinal developmental outcomes after traumatic brain injury in young children: are infants more vulnerable than toddlers? J Neurotrauma 2019;36(2):282–292; doi: 10.1089/neu.2018.5687 30019631 PMC6338576

[4] Gagner C, Landry-Roy C, Bernier A, et al. Behavioral consequences of mild traumatic brain injury in preschoolers. Psychol Med 2018;48(9):1551–1559; doi: 10.1017/S0033291717003221 29173217

[5] Larner SF, Hayes RL, McKinsey DM, et al. Increased expression and processing of caspase-12 after traumatic brain injury in rats. J Neurochem 2004;88(1):78–90; doi: 10.1046/j.1471-4159.2003.02141.x 14675152

[6] Begum G, Yan HQ, Li L, et al. Docosahexaenoic acid reduces ER stress and abnormal protein accumulation and improves neuronal function following traumatic brain injury. J Neurosci 2014;34(10):3743–3755; doi: 10.1523/JNEUROSCI.2872-13.2014 24599472 PMC6608987

[7] Larner SF, Hayes RL, Wang KK. Unfolded protein response after neurotrauma. J Neurotrauma 2006;23(6):807–829; doi: 10.1089/neu.2006.23.807 16774469

[8] Sokka AL, Putkonen N, Mudo G, et al. Endoplasmic reticulum stress inhibition protects against excitotoxic neuronal injury in the rat brain. J Neurosci 2007;27(4):901–908; doi: 10.1523/JNEUROSCI.4289-06.2007 17251432 PMC6672923

[9] Wu A, Ying Z, Gomez-Pinilla F. Omega-3 fatty acids supplementation restores mechanisms that maintain brain homeostasis in traumatic brain injury. J Neurotrauma 2007;24(10):1587–1595; doi: 10.1089/neu.2007.0313 17970622

[10] Dash PK, Hylin MJ, Hood KN, et al. Inhibition of eukaryotic initiation factor 2 alpha phosphatase reduces tissue damage and improves learning and memory after experimental traumatic brain injury. J Neurotrauma 2015;32(20):1608–1620; doi: 10.1089/neu.2014.3772 25843479 PMC4593880

[11] Wu F, Xu K, Liu L, et al. Vitamin B12 enhances nerve repair and improves functional recovery after traumatic brain injury by inhibiting ER stress-induced neuron injury. Front Pharmacol 2019;10:406; doi: 10.3389/fphar.2019.00406 31105562 PMC6491933

[12] Wang ZF, Gao C, Chen W, et al. Salubrinal offers neuroprotection through suppressing endoplasmic reticulum stress, autophagy and apoptosis in a mouse traumatic brain injury model. Neurobiol Learn Mem 2019;161:12–25; doi: 10.1016/j.nlm.2019.03.002 30851432

[13] Hood KN, Zhao J, Redell JB, et al. Endoplasmic reticulum stress contributes to the loss of newborn hippocampal neurons after traumatic brain injury. J Neurosci 2018;38(9):2372–2384; doi: 10.1523/JNEUROSCI.1756-17.2018 29386258 PMC5830522

[14] Smith DH, Uryu K, Saatman KE, et al. Protein accumulation in traumatic brain injury. Neuromolecular Med 2003;4(1–2):59–72; doi: 10.1385/NMM:4:1-2:59 14528053

[15] Hylin MJ, Holden RC, Smith AC, et al. Juvenile traumatic brain injury results in cognitive deficits associated with impaired endoplasmic reticulum stress and early tauopathy. Dev Neurosci 2018;40(2):175–188; doi: 10.1159/000488343 29788004 PMC6376969

[16] Hwang J, Qi L. Quality control in the endoplasmic reticulum: crosstalk between ERAD and UPR pathways. Trends Biochem Sci 2018;43(8):593–605; doi: 10.1016/j.tibs.2018.06.005 30056836 PMC6327314

[17] Meusser B, Hirsch C, Jarosch E, et al. ERAD: the long road to destruction. Nat Cell Biol 2005;7(8):766–772; doi: 10.1038/ncb0805-766 16056268

[18] Walter P, Ron D. The unfolded protein response: from stress pathway to homeostatic regulation. Science 2011;334(6059):1081–1086; doi: 10.1126/science.1209038 22116877

[19] Mori K. Tripartite management of unfolded proteins in the endoplasmic reticulum. Cell 2000;101(5):451–454; doi: 10.1016/s0092-8674(00)80855-7 10850487

[20] Yoshida H, Matsui T, Yamamoto A, et al. XBP1 mRNA is induced by ATF6 and spliced by IRE1 in response to ER stress to produce a highly active transcription factor. Cell 2001;107(7):881–891; doi: 10.1016/s0092-8674(01)00611-0 11779464

[21] Calfon M, Zeng H, Urano F, et al. IRE1 couples endoplasmic reticulum load to secretory capacity by processing the XBP-1 mRNA. Nature 2002;415(6867):92–96; doi: 10.1038/415092a 11780124

[22] Sitia R, Braakman I. Quality control in the endoplasmic reticulum protein factory. Nature 2003;426(6968):891–894; doi: 10.1038/nature02262 14685249

[23] Harding HP, Zhang Y, Ron D. Protein translation and folding are coupled by an endoplasmic-reticulum-resident kinase. Nature 1999;397(6716):271–274; doi: 10.1038/16729 9930704

[24] Vattem KM, Wek RC. Reinitiation involving upstream ORFs regulates ATF4 mRNA translation in mammalian cells. Proc Natl Acad Sci U S A 2004;101(31):11269–11274; doi: 10.1073/pnas.0400541101 15277680 PMC509193

[25] Sen T, Saha P, Gupta R, et al. Aberrant ER stress induced neuronal-IFNβ elicits white matter injury due to microglial activation and t-cell infiltration after TBI. J Neurosci 2020;40(2):424–446; doi: 10.1523/JNEUROSCI.0718-19.2019 31694961 PMC6948950

[26] Zhang Z, Saraswati M, Koehler RC, et al. A new rabbit model of pediatric traumatic brain injury. J Neurotrauma 2015;32(17):1369–1379; doi: 10.1089/neu.2014.3701 25758339 PMC4543485

[27] Zhang Z, Rasmussen L, Saraswati M, et al. Traumatic injury leads to inflammation and altered tryptophan metabolism in the juvenile rabbit brain. J Neurotrauma 2018;36(1):74–86; doi: 10.1089/neu.2017.5450 PMC12430859 30019623

[28] Zhang Z, Ishrat S, O'Bryan M, et al. Pediatric traumatic brain injury causes long-term deficits in adult hippocampal neurogenesis and cognition. J Neurotrauma 2020;37(14):1656–1667; doi: 10.1089/neu.2019.6894 32079496

[29] Almanza A, Carlesso A, Chintha C, et al. Endoplasmic reticulum stress signalling—from basic mechanisms to clinical applications. FEBS J 2019;286(2):241–278; doi: 10.1111/febs.14608 30027602 PMC7379631

[30] Novoa I, Zeng H, Harding HP, et al. Feedback inhibition of the unfolded protein response by GADD34-mediated dephosphorylation of eIF2alpha. J Cell Biol 2001;153(5):1011–1022; doi: 10.1083/jcb.153.5.1011 11381086 PMC2174339

[31] Vicencio-Rosas E, Perez-Guille MG, Flores-Perez C, et al. Buprenorphine and pain treatment in pediatric patients: an update. J Pain Res 2018;11:549–559; doi: 10.2147/JPR.S153903 29588613 PMC5859905

[32] National Research Council (U.S.). Committee for the Update of the Guide for the Care and Use of Laboratory Animals, Institute for Laboratory Animal Research (U.S.), National Academies Press (U.S.). Guide for the Care and Use of Laboratory Animals. National Academies Press: Washington, DC; 2011.

[33] Plein LM, Rittner HL. Opioids and the immune system—friend or foe. Br J Pharmacol 2018;175(14):2717–2725; doi: 10.1111/bph.13750 28213891 PMC6016673

[34] Kim MS, Cheong YP, So HS, et al. Protective effects of morphine in peroxynitrite-induced apoptosis of primary rat neonatal astrocytes: potential involvement of G protein and phosphatidylinositol 3-kinase (PI3 kinase). Biochem Pharmacol 2001;61(7):779–786; doi: 10.1016/s0006-2952(01)00541-x 11274962

[35] Zhang C, Wang C, Ren J, et al. Morphine protects spinal cord astrocytes from glutamate-induced apoptosis via reducing endoplasmic reticulum stress. Int J Mol Sci 2016;17(10):1523; doi: 10.3390/ijms17101523 27783050 PMC5085616

[36] Carvallo L, Lopez L, Che FY, et al. Buprenorphine decreases the CCL2-mediated chemotactic response of monocytes. J Immunol 2015;194(7):3246–3258; doi: 10.4049/jimmunol.1302647 25716997 PMC4369415

[37] Ryu J, Stone P, Lee S, et al. Buprenorphine alters microglia and astrocytes acutely following diffuse traumatic brain injury. Sci Rep 2021;11(1):8620; doi: 10.1038/s41598-021-88030-z 33883663 PMC8060410

[38] Hamood Y, Abdullah M, El Ghoul H, et al. Sex specific effects of buprenorphine on behavior, astrocytic opioid receptor expression and neuroinflammation after pediatric traumatic brain injury in mice. Brain Behav Immun Health 2022;22:100469; doi: 10.1016/j.bbih.2022.100469 35620644 PMC9127176

[39] Semple BD, Carlson J, Noble-Haeusslein LJ. Pediatric rodent models of traumatic brain injury. Methods Mol Biol 2016;1462:325–343; doi: 10.1007/978-1-4939-3816-2_18 27604726

[40] Gutierrez IL, Gonzalez-Prieto M, Garcia-Bueno B, et al. Alternative method to detect neuronal degeneration and amyloid beta accumulation in free-floating brain sections with Fluoro-Jade. ASN Neuro 2018;10:1759091418784357; doi: 10.1177/1759091418784357 29950099 PMC6043921

[41] Wu J, Vogel T, Gao X, et al. Neuroprotective effect of dexmedetomidine in a murine model of traumatic brain injury. Sci Rep 2018;8(1):4935; doi: 10.1038/s41598-018-23003-3 29563509 PMC5862953

[42] Zhang Z, Bassam B, Thomas AG, et al. Maternal inflammation leads to impaired glutamate homeostasis and up-regulation of glutamate carboxypeptidase II in activated microglia in the fetal/newborn rabbit brain. Neurobiol Dis 2016;94:116–128; doi: 10.1016/j.nbd.2016.06.010 27326668 PMC5394739

[43] Hodeify R, Megyesi J, Tarcsafalvi A, et al. Gender differences control the susceptibility to ER stress-induced acute kidney injury. Am J Physiol Renal Physiol 2013;304(7):F875–F882; doi: 10.1152/ajprenal.00590.2012 23364800 PMC3625845

[44] Rossetti CL, de Oliveira Costa HM, Barthem CS, et al. Sexual dimorphism of liver endoplasmic reticulum stress susceptibility in prepubertal rats and the effect of sex steroid supplementation. Exp Physiol 2019;104(5):677–690; doi: 10.1113/EP087518 30821070

[45] Oyadomari S, Mori M. Roles of CHOP/GADD153 in endoplasmic reticulum stress. Cell Death Differ 2004;11(4):381–389; doi: 10.1038/sj.cdd.4401373 14685163

[46] Krajewska M, Xu L, Xu W, et al. Endoplasmic reticulum protein BI-1 modulates unfolded protein response signaling and protects against stroke and traumatic brain injury. Brain Res 2011;1370:227–237; doi: 10.1016/j.brainres.2010.11.015 21075086 PMC3019258

[47] Rajesh K, Krishnamoorthy J, Kazimierczak U, et al. Phosphorylation of the translation initiation factor eIF2α at serine 51 determines the cell fate decisions of Akt in response to oxidative stress. Cell Death Dis 2015;6(1):e1591; doi: 10.1038/cddis.2014.554 25590801 PMC4669752

[48] Chakrabarti S, Liu NJ, Gintzler AR. Formation of mu-/kappa-opioid receptor heterodimer is sex-dependent and mediates female-specific opioid analgesia. Proc Natl Acad Sci U S A 2010;107(46):20115–20119; doi: 10.1073/pnas.1009923107 21041644 PMC2993367

[49] Tan M, Wang H, Gao C, et al. Agonists specific for κ-opioid receptor induces apoptosis of HCC cells through enhanced endoplasmic reticulum stress. Front Oncol 2022;12:844214; doi: 10.3389/fonc.2022.844214 35433440 PMC9008754

[50] Casas-Tinto S, Zhang Y, Sanchez-Garcia J, et al. The ER stress factor XBP1s prevents amyloid-beta neurotoxicity. Hum Mol Genet 2011;20(11):2144–2160; doi: 10.1093/hmg/ddr100 21389082 PMC3090193

[51] Waldherr SM, Strovas TJ, Vadset TA, et al. Constitutive XBP-1s-mediated activation of the endoplasmic reticulum unfolded protein response protects against pathological tau. Nat Commun 2019;10(1):4443; doi: 10.1038/s41467-019-12070-3 31570707 PMC6768869

[52] Johnson VE, Stewart W, Smith DH. Traumatic brain injury and amyloid-beta pathology: a link to Alzheimer's disease? Nat Rev Neurosci 2010;11(5):361–370; doi: 10.1038/nrn2808 20216546 PMC3979339

[53] Edwards G III, Zhao J, Dash PK, et al. Traumatic brain injury induces tau aggregation and spreading. J Neurotrauma 2020;37(1):80–92; doi: 10.1089/neu.2018.6348 31317824 PMC6921297

[54] Sundermann EE, Panizzon MS, Chen X, et al.; Alzheimer's Disease Neuroimaging Initiative. Sex differences in Alzheimer's-related Tau biomarkers and a mediating effect of testosterone. Biol Sex Differ 2020;11(1):33; doi: 10.1186/s13293-020-00310-x 32560743 PMC7304096

[55] Oveisgharan S, Arvanitakis Z, Yu L, et al. Sex differences in Alzheimer's disease and common neuropathologies of aging. Acta Neuropathol 2018;136(6):887–900; doi: 10.1007/s00401-018-1920-1 30334074 PMC6279593

[56] Tsiknia AA, Edland SD, Sundermann EE, et al.; Alzheimer's Disease Neuroimaging Initiative. Sex differences in plasma p-tau181 associations with Alzheimer's disease biomarkers, cognitive decline, and clinical progression. Mol Psychiatry 2022;27(10):4314–4322; doi: 10.1038/s41380-022-01675-8 35768637 PMC9718670

[57] Buckley RF, Mormino EC, Rabin JS, et al. Sex differences in the association of global amyloid and regional tau deposition measured by positron emission tomography in clinically normal older adults. JAMA Neurol 2019;76(5):542–551; doi: 10.1001/jamaneurol.2018.4693 30715078 PMC6515599

[58] Mondello S, Guedes VA, Lai C, et al. Sex differences in circulating t-tau trajectories after sports-concussion and correlation with outcome. Front Neurol 2020;11:651; doi: 10.3389/fneur.2020.00651 32733367 PMC7358531

